# Identification of critical brain regions for autism diagnosis from fMRI data using explainable AI: an observational analysis of the ABIDE dataset

**DOI:** 10.1016/j.eclinm.2025.103452

**Published:** 2025-09-18

**Authors:** Suryansh Vidya, Kush Gupta, Amir Aly, Andy Wills, Emmanuel Ifeachor, Rohit Shankar

**Affiliations:** University of Plymouth, Drake Circus, Plymouth, PL4 8AA, UK

**Keywords:** Biomarkers, Deep learning, Explainable AI, Functional connectivity, Functional MRI (fMRI), Interpretability, Magnetic resonance imaging, Neuroimaging, Remove and Retrain (ROAR), Sparse autoencoders

## Abstract

**Background:**

Diagnosis of autism spectrum disorder (ASD) significantly improves quality of life, yet current diagnostic practices rely on subjective behavioural assessments. While machine learning models using functional Magnetic Resonance Imaging (fMRI) show promise for objective diagnosis, existing approaches have critical limitations. High-accuracy models often neglect interpretability, increasing clinical distrust, while interpretable approaches frequently suffer from low accuracy and lack neuroscientific validation of identified biomarkers. Furthermore, no studies have systematically benchmarked which interpretability methods are most effective for fMRI data, hindering the translation of Artificial intelligence (AI) findings into clinical practice.

**Methods:**

In this observational study, we used the Autism Brain Imaging Data Exchange I (ABIDE I) dataset comprising 884 participants (408 with ASD; 476 controls with typical development) aged 7–64 years from five countries (Belgium, Germany, Ireland, the Netherlands, and the USA) across 17 international sites after applying mean framewise displacement (FD) filtering (>0.2 mm). Data were collected at the respective sites prior to the release of the ABIDE I dataset in August 2012. We described an explainable deep learning (DL) pipeline using a Stacked Sparse Autoencoder (SSAE) with a softmax classifier, trained on functional connectivity data. We systematically benchmarked seven interpretability methods using the Remove And Retrain (ROAR) technique and cross-validated our findings across three preprocessing pipelines. Critically, we validated identified biomarkers against independent neuroscientific literature from genetic, neuroanatomical, and functional studies.

**Findings:**

Filtering head movement (FD > 0.2 mm) increased classification accuracy from 91% to 98.2% (F1-score: 0.97), achieving state-of-the-art performance. ROAR analysis revealed gradient-based methods, particularly Integrated Gradients, as most reliable for fMRI interpretation. The model consistently identified visual processing regions (calcarine sulcus, cuneus) as critical for ASD classification. These findings aligned with independent genetic studies and neuroimaging research, confirming our model captured genuine neurobiological ASD markers rather than overfitting to dataset artifacts.

**Interpretation:**

We addressed three key research gaps by developing a highly accurate, interpretable ASD classification model, establishing a benchmarking framework for interpretability methods in the fMRI modality, and validating our identified biomarkers against established neuroscience literature. The consistent importance of visual processing regions suggests a fundamental biomarker potentially present across the ASD spectrum, offering new directions for diagnostic biomarker research, though translation to individual-level clinical applications requires further development.

**Funding:**

This work was supported by the Engineering and Physical Sciences Research Council, UK [EP/W524554/1].

**Research in context**

**Evidence before this study**

We searched PubMed, arXiv, IEEE Xplore, and Google Scholar for studies published in English between Jan 1, 2010, and June 31, 2025, using the terms “autism spectrum disorder”, “ASD”, “functional connectivity”, “fMRI”, “machine learning”, “deep learning”, and “interpretability”. With Autism Spectrum Disorder (ASD) diagnoses increasing dramatically from 1% to 3% of the population over two decades and diagnostic waiting lists exceeding 200,000 individuals in the UK alone, objective diagnostic tools are urgently needed. The search yielded 2847 studies. After screening for relevance to autism classification using fMRI data and interpretability methods, we identified three critical research gaps in existing literature: 1) High-accuracy models for ASD classification (>90%) often neglect interpretability, creating clinical “black boxes” that fail to explain which neural connections drive their decisions; 2) No systematic benchmarking framework exists to determine which interpretability methods are most effective specifically for fMRI-based functional connectivity data, hindering the identification of optimal approaches for this domain; 3) Studies that incorporate interpretability typically suffer from multiple methodological limitations including low accuracy (<80%), small sample sizes (<100 participants), employment of only single interpretability method without comparative assessment of alternatives, or failure to validate identified regions against established neuroscientific literature, raising questions about their reliability and clinical utility.

**Added value of this study**

Our study addresses these three research gaps through corresponding key contributions: First, we developed a highly accurate yet interpretable deep learning model (98.2% accuracy, F1-score: 0.97) for ASD classification using functional connectivity data from the Autism Brain Imaging Data Exchange, demonstrating that the accuracy interpretability trade-off can be overcome. Second, we addressed the methodological limitations of previous interpretability studies by employing a large sample size, achieving high accuracy, systematically comparing seven different interpretability approaches, and validating identified biomarkers against independent neuroscientific literature. Third, we established the first comprehensive benchmarking framework for evaluating interpretability methods in functional Magnetic Resonance Imaging (fMRI) based ASD classification using the Remove And Retrain (ROAR) technique, identifying that gradient-based methods, particularly Integrated Gradients, most reliably identify discriminative features. Our model consistently highlighted visual processing regions (calcarine sulcus, cuneus) across different preprocessing pipelines, with these findings independently validated by genetic studies identifying the same Brodmann areas in autism, confirming these as genuine neural biomarkers rather than dataset artifacts.

**Implications of all the available evidence**

By uniting high classification performance with systematically validated interpretability, our research provides a comprehensive framework for leveraging deep learning in ASD diagnosis while maintaining clinical trust in AI-based diagnostic tools through transparency. The consistent identification of visual processing regions as key discriminative features across preprocessing pipelines suggests a fundamental neurobiological signature that may be present across the ASD spectrum, potentially representing an important biomarker for objective diagnosis across age ranges. This finding aligns with emerging evidence from multiple research disciplines indicating that differences in early visual processing may contribute to broader social and behavioural symptoms in ASD. Our benchmarking framework establishes a methodological standard for future neuroimaging studies, advancing both the technical implementation and clinical translation of explainable AI for fMRI data. These results offer new directions for diagnostic biomarker research while demonstrating how systematic interpretability validation can bridge the gap between computational performance and meaningful clinical insights.

## Introduction

Autism Spectrum Disorder (ASD) is a complex neurodevelopmental condition characterised by impairments in social communication, interaction, and restricted, repetitive behaviours.^1^ The prevalence of ASD has increased dramatically in recent decades, from approximately 1% to around 3% of the population, representing a 787% surge in diagnoses over the last 20 years.^2^, ^3^, ^4^ This dramatic increase, largely driven by adult diagnosis particularly in women,^2^^,^^3^^,^^5^ combined with labour-intensive assessment procedures, has created a diagnostic crisis with substantial waiting lists exceeding 200,000 individuals in the UK alone.^5^ Research has shown that intervention of ASD can enhance Intelligence Quotient (IQ) scores by up to 18 points, significantly improve language skills,^6^ and lead to a better Quality of Life (QoL) for individuals with ASD especially if they are diagnosed early (age 2–5 years).^7^

Current clinical diagnostic practices for ASD rely heavily on behavioural analysis and clinical history, methods that present significant limitations for diagnosis. These approaches depend on identifying abnormal communication and social behaviours that often do not fully emerge until the condition is well established.^8^ Additionally, behavioural assessments are vulnerable to clinician biases, which have historically led to under-diagnosis in certain populations, particularly females.^9^ These limitations underscore the urgent need for objective, biologically-based diagnostic markers that can enable more efficient and accurate identification of ASD across diverse populations.

Resting-state functional Magnetic Resonance Imaging (rs-fMRI) has emerged as a promising approach for identifying objective biomarkers of ASD. Studies of rs-fMRI data have shown abnormalities in functional connectivity patterns among individuals with ASD,^10^ often manifesting as local over-connectivity combined with long-range under-connectivity.^11^, ^12^, ^13^ Key regions implicated include the frontal cortex,^14^ temporal cortex,^15^ amygdala,^16^ and cerebellum.^17^ More recent studies have also implicated primary sensory cortex, with genetic research finding that Brodmann Area (BA) 17 (primary visual cortex) showed the most abnormal findings in ASD.^18^ This Nature study found abnormalities in transcriptomics across the cortex, potentially related to a dimension of neural density decreasing from posterior to anterior.^18^ Abnormalities in large-scale networks such as the Default Mode Network (DMN) and Salience Network (SN) have also been linked to ASD.^19^^,^^20^ Furthermore, a significant challenge in biomarker development is the “idiosyncratic brain” concept, where functional connectivity patterns vary widely among individuals with ASD, complicating the search for universal biomarkers and necessitating analytical approaches.

Deep learning has demonstrated promise for analysing complex fMRI data and identifying subtle patterns that distinguish individuals with ASD from typically developing controls. Several studies have reported classification accuracies exceeding 90%.^21^^,^^22^ However, as these models achieve higher accuracy, the need for interpretability becomes increasingly critical for clinical acceptance and neurobiological validation.

Recent advances in explainable AI for medical imaging have shown promise across various domains, including cervical cancer screening using gradient-based methods like GradCAM++ and Layer-wise Relevance Propagation,^23^ melanoma detection employing GradCAM variants,^24^ and glaucoma identification using visualisation techniques.^25^ These applications predominantly focus on image-based modalities with visible features, and their interpretability approaches have not been validated for functional connectivity data in neuroimaging applications.

Critically, three research gaps persist in the current research landscape. First, high-accuracy models for ASD diagnosis often operate as “black boxes”, providing little insight into which brain regions or connections drive their decisions.^26^^,^^27^ Second, no systematic benchmarking exists to determine which interpretability approaches are most effective for fMRI-based functional connectivity data. Third, studies that prioritise interpretability frequently suffer from lower accuracy (<80%), rely on small sample sizes, employ only one interpretability method without comparative assessment, or fail to validate identified brain regions against established neuroscientific literature.

To address these three gaps, we describe a deep learning framework that achieves both high classification accuracy and robust interpretability for ASD detection using rs-fMRI data from the ABIDE I dataset. Our approach combines a Stacked Sparse Autoencoder (SSAE) with a softmax classifier, incorporating unsupervised pre-training and supervised fine-tuning to capture subtle but distinctive patterns in functional connectivity. We systematically benchmark seven different interpretability methods using the ROAR technique^28^ to identify which approach most reliably highlights discriminative features in functional connectivity data and we validate our findings against independent genetic, neuroanatomical, and functional studies of ASD to ensure that our model captures genuine neurobiological markers rather than dataset-specific artifacts.

This study addresses three primary research questions corresponding to the identified gaps: (RQ1) Can we develop a highly accurate yet interpretable deep learning model for ASD classification using rs-fMRI data? (RQ2) Which interpretability methods are most reliable for functional connectivity analysis, and how can we systematically benchmark their performance? (RQ3) What regions in the brain are biomarkers for ASD and do the regions align with established neurobiological findings in ASD from independent research domains? Beyond advancing technical methods, our work aims to contribute meaningfully to clinical practice by enhancing our understanding of the neurobiological basis of ASD, potentially supporting more efficient and objective diagnosis and more targeted interventions. Successful application of this research could ultimately lead to improved diagnostic accessibility, reduced healthcare system burden, and enhanced quality of life for individuals with ASD and their families.

## Methods

### Study design and ethics

**Fig. 1:**
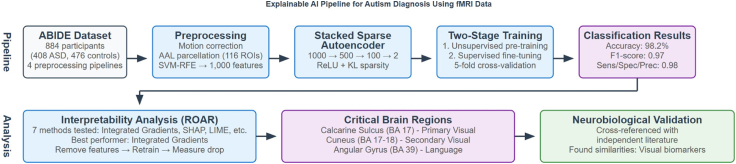
Explainable AI pipeline for autism diagnosis using resting state functional magnetic resonance imaging (rs-fMRI) Data.

Fig. 1 presents an overview of our complete methodological pipeline, illustrating the progression from data preprocessing through model training to interpretability analysis and validation. Since the ABIDE I dataset is a public, anonymised dataset with no protected health information included, we did not require any ethical approval as per the dataset's data usage agreement. Data collection of the ABIDE I dataset was performed in accordance with Health Insurance Portability and Accountability (HIPAA) guidelines and 1000 Functional Connectomes Project/International Neuroimaging Data-sharing Initiative protocols.

### Data collection and preprocessing

#### Dataset

This study used the ABIDE I dataset,^29^ which comprises resting-state fMRI data from individuals aged 7 to 64 years with autism (n = 408) and with typical development (n = 476) from 17 international imaging sites (Table 1), collected prior to its release in August 2012. The participants with ASD have varying classic total Autism Diagnostic Observation Schedule^30^ (ADOS) scores, derived from the sum of communication and social interaction subscores, representing a spectrum of symptom severity. The sample exhibits a significant gender imbalance (85.2% male) that reflects the diagnostic practices and understanding of ASD prevalence at the time of data collection.^31^ While this ratio was considered representative when ABIDE I was assembled, current evidence suggests autism is under-diagnosed in females due to symptom masking and diagnostic bias.^32^

**Table 1:** Information for the included participants from Autism Brain Imaging Data Exchange (ABIDE) dataset for each individual site.

Site	Individuals with autism spectrum disorder	Typically developing controls
Carnegie Mellon University, USA	3	2
California Institute of Technology (Caltech), USA	19	18
Kennedy Krieger Institute, USA	12	27
University of Leuven, Belgium	27	34
Ludwig Maxomilians University Munich (Maxmun), Germany	18	24
NYU Langone Medical Center, USA	73	98
Oregon Health and Science University, USA	12	11
Olin Institute of Living at Hartford Hospital, USA	14	11
University of Pittsburgh School of Medicine, USA	22	23
Social Brain Lab, the Netherlands	14	12
San Diego State University, USA	12	21
Stanford University, USA	17	19
Trinity Centre for Health Sciences, Ireland	21	23
University of California Los Angeles, USA	36	39
University of Michigan, USA	48	65
Utah school of medicine, USA	38	23
Yale child study center, USA	22	26

#### Preprocessing

Preprocessing pipelines help harmonise the data from potential confounders such as site effects, age range. The Preprocessed Connectomes Project (PCP) openly shares ABIDE I data preprocessed via four pipelines—the Connectome Computation System (CCS),^33^ the Configurable Pipeline for the Analysis of Connectomes (CPAC),^34^ the Data Processing Assistant for Resting-State fMRI (DPARSF),^35^ and the Neuroimaging Analysis Kit (NIAK).^36^ These pipelines are intended to mitigate centre-specific artifacts. Because no single pipeline is universally optimal, we trained our model on all four to compare performance.^37^ Additionally, analysing data from multiple pipelines helps us identify the core regions that remain consistent regardless of preprocessing choices, ensuring that any discovered biomarkers are pipeline independent. Full details on these pipelines are available in the PCP documentation.^37^

Each pipeline applies standard low-level preprocessing steps, such as brain extraction, realignment (motion correction), and slice timing correction.^34^ NIAK normalises functional data to template space before calculating connectivity measures, while the other pipelines (DPARSF, CPAC, CCS) perform calculations in subject-native space before spatial normalisation. Further, there has been significant debate in the literature about whether mean Framewise Displacement (FD) filtering influences ASD classification performance. FD is a measure of how much head movement occurs during the MRI scan. Some influential studies state that a FD value > 0.2 mm corrupts the rs-fMRI data,^38^^,^^39^ leading to decreased accuracy, whereas another study^40^ contends that head motion does not appreciably affect performance. We conducted experiments both with and without FD filtering to clarify this methodological issue.

Applying an FD threshold of 0.2 mm yielded 884 usable samples (408 ASD, 476 controls [typical development]). These were drawn from multiple international sites and scanners.

#### Parcellation

Brain images were then parcellated with the Automated Anatomical Labelling (AAL) atlas, which divides the brain into 116 Regions of Interest (ROIs). We selected the AAL atlas for its balance of anatomical detail and manageable complexity, as recommended in similar ASD studies.^22^^,^^41^ This produced an *N* × *t* data array (where *N* = 116 ROIs, *t* is the temporal dimension) for each of the 884 participants, providing the foundation for subsequent feature extraction.

### Feature extraction

To reveal the patterns of coordination between brain regions, each rs-fmri time-series was converted into a functional connectivity matrix (FCM) by calculating Pearson's correlation coefficient *R* (*i, j*) between the intensity time series from every pair of ROIs. We then applied Fisher's Z transformation to normalise these correlation coefficients. Finally, to reduce redundancy, we flattened the lower triangular of each symmetric FCM (116 × 116), yielding 6670 features per sample.

Creating a feature vector from the FCM has been thoroughly researched and used in DL models, however, when using them directly for autism diagnosis, the DL models peaked at an accuracy of 66.8%.^42^^,^^43^ This implies that these DL models suffer from the high-dimensionality and low number of samples problem. To efficiently use DL models, feature dimensionality must be reduced, or sample size must be increased. However, increasing sample size is infeasible due to reasons including high MRI costs and difficulty in participant recruitment.

To address this, we employed Support Vector Machine with Recursive Feature Elimination (SVM-RFE) for dimensionality reduction. We chose SVM-RFE over unsupervised methods like principal component analysis (PCA) for its task-specific feature selection and proven performance in similar studies,^22^ ultimately reducing the feature set from 6670 to 1000 features per sample. This mitigates the computational burden and helps the model focus on the most discriminative functional connections.

### Deep learning model

**Fig. 2:**
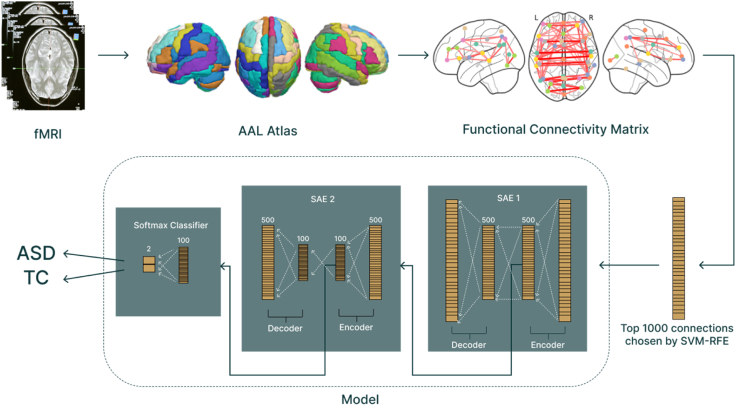
The overall architecture of the model, incorporating both the Stacked Sparse Autoencoder (SSAE) and softmax classifier.

This study utilises a DL architecture (Fig. 2) comprising a SSAE combined with a softmax classifier. Autoencoders learn a compressed representation of the input, and the *sparse* constraint encourages focus on only the most distinctive biomarkers, which is important for high-dimensional functional connectivity data.

Our choice of a two-layer SSAE with ReLU activation and Kullback-Leibler (KL) divergence sparsity penalty is motivated by both practical and empirical considerations.^22^^,^^43^ KL divergence is a measure from information theory that quantifies how different one probability distribution is from another. We found that two hidden layers strike an optimal balance between expressive power and training complexity, especially given our sample size. Hyperparameter tuning indicated that deeper networks did not improve performance, so this design effectively balances model expressiveness with the risk of overfitting. ReLU activations have shown promising performance in similar high-dimensional neuroimaging tasks, avoiding saturation issues observed in sigmoid/tanh functions. Crucially, the KL divergence term enforces neuron-level sparsity, highlighting discriminative features while mitigating overfitting,^42^ as captured by the following loss function *L* ($x,\hat{x}$) between input *x* and its reconstruction $\hat{x}$:

(1) $$L\left(x,\hat{x}\right)=MSE+\beta \;\times \;KL\left(\rho ∥{\hat{\rho }}_{j}\right)$$

where *MSE* is the mean squared error between input *x* and its reconstruction $\hat{x}$, *ρ* is the desired sparsity, ${\hat{\rho }}_{j}$ is the average activation of the *j*-th hidden neuron, and *β* controls the weight of the sparsity penalty.

Our SSAE consisted of two autoencoder layers that used the ReLU activation function. The first reduces 1000 input features to 500, and the second further compresses to 100 neurons. The output was then fed into a softmax classifier that was trained using cross-entropy loss for ASD/control (typical development) classification. This is visualised in Fig 2.

### Training and evaluation

The model was trained in two stages; unsupervised pre training followed by supervised fine-tuning-to combine the strengths of both approaches. We used stratified 5-fold cross-validation to balance computational cost with variance reduction, given our dataset of 884 samples. Within each fold, the data were split into 64% training, 16% validation, and 20% testing. A public repository with our code will be provided to facilitate reproducibility.

The SSAE underwent unsupervised pre-training using the greedy layer-wise training approach.^44^ This sequential approach allows each autoencoder layer to learn increasingly abstract representations of the input data. AE 1 was trained first and its compressed hidden layer representation then served as input for training AE 2. We employed the Adam optimiser^45^ throughout, chosen for its adaptive learning rate and rapid convergence.

Once pretraining was complete, the encoder layers were combined with a softmax classifier. In contrast to approaches that fine-tune only the classifier, we fine-tuned both the encoder and classifier parameters together to enhance discrimination between ASD and typical development (controls).^42^^,^^43^^,^^46^ This supervised phase again used Adam, with hyperparameters adjusted to avoid overfitting while refining the learned representations for classification. The following hyperparameters were used: pre-training 50 epochs, learning rate of 0.001, weight decay of 0.0001, batch size of 128, KL Divergence parameters: *ρ* = 0.2, *β* = 2. Fine-tuning 50 epochs, learning rate of 0.0001, weight decay of 0.0001, batch size of 128.

We report accuracy, sensitivity, specificity, precision, and F1-scores, each averaged across the five folds and compare the results with literature (Table 2). This ensures a thorough assessment of model performance beyond simple accuracy. Additionally, we monitored training and validation losses to detect overfitting, providing insight into whether the model surmounts typical performance limitations reported in prior ASD classification work.

**Table 2:** Accuracy comparison with studies using resting state functional magnetic resource imaging (rs-fMRI) data.

Model	Accuracy (%)
Wang et al. (2019)^22^	93.5
Pavithra et al. (2023)^47^	85
Bhandage et al. (2023)^48^	92.4
Wadhere et al. (2023)^49^	88.1
Herath et al. (2024)^50^	97.8
Kang et al. (2025)^51^	83.5
**Our model**	**98.2**

Several post-hoc analyses were conducted in response to peer review feedback, including: assessment of metric variance across cross-validation folds, detailed comparison of preprocessing pipeline performance differences, analysis of demographic representation limitations, and evaluation of computational requirements for clinical translation.

### Feature analysis and interpretation

#### Interpretability methods

We evaluated seven approaches (gradient- and perturbation-based) to capture different perspectives of feature importance. Specifically, we used Integrated Gradients,^52^ LIME,^53^ SHAP,^54^ DeepLift,^55^ DeepLiftShap,^56^ GradientShap,^54^ and GuidedBackprop.^57^ Gradient-based methods (Integrated Gradients, DeepLift, DeepLiftShap, GradientShap) directly quantify how changes in input features affect predictions. LIME employs local perturbations to approximate model decisions, whereas SHAP uses a game-theoretic framework to attribute feature importance. Guided Backprop highlights salient input features via positive gradients. Because each technique might emphasise different aspects of the model, benchmarking them was essential for trustworthy interpretation.

#### Remove and Retrain (ROAR)

We systematically compared these methods using the ROAR framework.^28^ First, each interpretability technique ranks the input features by importance. Next, we replace the most influential features (above a chosen threshold) with an uninformative value (zero). Zero was chosen as the uninformative value because the input features are correlation coefficients, and zero correlation indicates that no positive or negative relationship exists between the two variables. Hence, zero is uninformative in the context of functional connectivity. We evaluated feature removal at multiple thresholds [0.01, 0.05, 0.1, 0.2, 0.3, 0.4, 0.5, 0.6, 0.7, 0.8, 0.9, 0.95, 0.99] representing the proportion of top-ranked features replaced. Finally, for each threshold, we retrain the model from scratch on this modified dataset and measure the drop in classification performance. Larger performance decreases indicate that the removed features were indeed critical for the model's predictive capability, and we can use this to identify which interpretability technique is most reliable (steepest drop in accuracy).

#### Important Regions of Interest (ROIs)

**Fig. 3:**
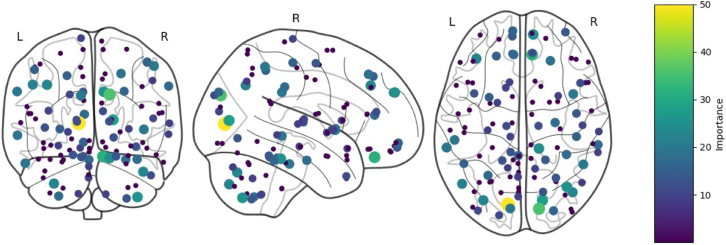
Important Regions of Interest (ROIs) identified by the Integrated Gradients approach for the Data Processing Assistant for Resting-State fMRI (DPARSF) pipeline, with colour intensity indicating importance level. The three views show: posterior/back view (left), right lateral view (middle), and superior/top view (right).

Using the interpretability technique that was identified as most reliable, we determine which features (correlation coefficients between brain regions) were important and aggregated them at the region level. Specifically, we identified how often ROIs appeared among top-weighted features and then mapped these important ROIs to their anatomical labels, as seen in Fig. 3. We then mapped the features across the different pipelines to Brodmann Areas (BA) (Table 3) and analysed the overlapping features. This analysis enabled us to pinpoint the key brain areas driving ASD classification and to compare our findings against established ASD-related neurobiology. This whole process can be seen in the.

**Table 3:** The top Regions Of Interest (ROIs) identified by each pipeline in decreasing order of importance alongside its corresponding Brodmann Area.

Connectome Computation System (CCS)	Brodmann area	Configurable Pipeline for the Analysis of Connectomes (CPAC)	Brodmann area	Data Processing Assistant for Resting-State fMRI (DPARSF)	Brodmann area
Calcarine_L	17	Calcarine_R	17	Calcarine_L	17
Occipital_Sup_R	17	Cuneus_R	17, 18	Rectus_R	11, 12
Occipital_Mid_R	17	Occipital_Sup_R	17	Cuneus_R	17, 18
Cuneus_R	17, 18	Frontal_Inf_Tri_R	44, 45	Temporal_Mid_L	21
Angular_R	39	Angular_L	39	Cerebelum_Crus1_R	–
Cerebelum_8_L	–	Cerebelum_8_R	–	Calcarine_R	17
Thalamus_R	41, 42	Cerebelum_Crus2_L	–	Cerebelum_3_R	–
Calcarine_R	17	Calcarine_L	17	Temporal_Inf_L	20
Insula_R	13, 16	Lingual_L	17	Occipital_Sup_R	17
SupraMarginal_R	40	ParaHippocampal_L	34	Frontal_Sup_Medial_R	11, 12
Parietal_Inf_L	5	Precentral_R	4	Frontal_Sup_L	11, 12
Heschl_R	41	Thalamus_L	41, 42	Angular_L	39

### Role of the funding source

This study did not receive funding.

## Results

The model achieved state-of-the-art performance on the ABIDE I dataset, with a maximum accuracy of 98.2% ± 1.2% across three different preprocessing pipelines, exceeding the previous benchmark of 97.82% (Table 2). Our overall metrics were equally strong (sensitivity: 0.98 ± 0.01, specificity: 0.98 ± 0.01, precision: 0.98 ± 0.01, F1-score: 0.97 ± 0.01). Below, we detail performance and interpretability results. A standard desktop with Intel i5-12400 processor, 32 GB RAM, AMD RX 6800 GPU was used for training, cross-validation, ROAR analysis. The training, cross-validation, and ROAR analysis took ∼2 h.

### Model performance

Our model demonstrated that high accuracy and interpretability can be achieved simultaneously (Research Question 1). Filtering scans with FD > 0.2 mm^38^^,^^39^ boosted accuracy from 91% to 98.2%. The model achieved consistent performance across cross-validation folds (mean accuracy: 98.2% ± 1.2%). DPARSF achieved highest accuracy (98.2%), followed by CPAC (97.6%), CCS (97.2%), and NIAK (93.1%). NIAK was dropped from feature analysis due to its worse performance and analysis was only done on results from the CCS, CPAC and DPARSF pipelines.

**Fig. 4:**
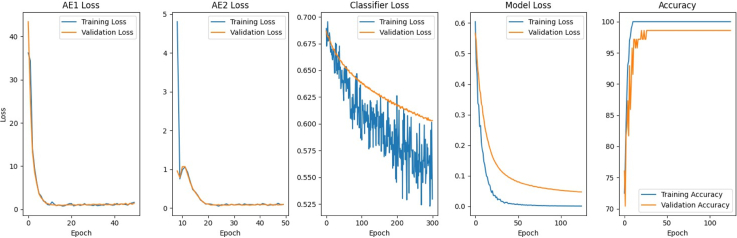
Loss graphs during first K-Fold. AE1, AE2, and classifier loss represent reconstruction loss during unsupervised pre-training. Model loss and accuracy refer to the supervised fine-tuning stage.

To check for overfitting, we monitored training and validation loss curves (Fig. 4) which showed stable convergence without divergence. Observing Fig. 4, the autoencoders (AE1 and AE2 loss) consistently reconstruct the data during unsupervised pre-training. In the subsequent supervised fine-tuning phase, when diagnostic labels are introduced, cross-entropy loss (model loss) drops steadily for both training and validation sets.

### Interpretability method benchmarking

**Fig. 5:**
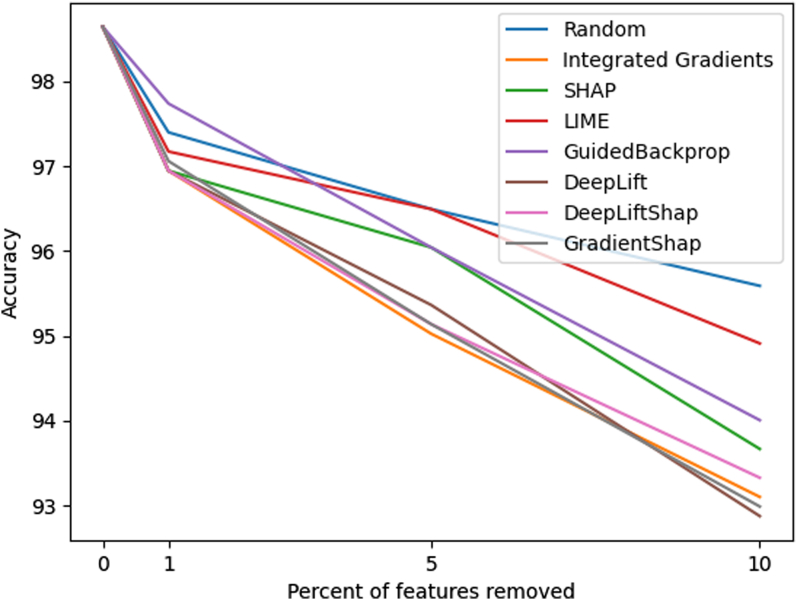
Remove and Retrain (ROAR) Analysis showing the impact of feature removal on model accuracy.

The ROAR analysis revealed that gradient-based methods, particularly Integrated Gradients, most reliably identify discriminative features for functional connectivity data (Research Question 2). Similarly, the DeepLift, DeepLiftShap, and GradientShap methods (all gradient-based methods) performed well and showed the steepest drops in accuracy when the initial features were removed (Fig. 5). Since the Integrated Gradients approach showed the steepest drop in accuracy, it was therefore assumed to be the most reliable method. Notably, the LIME approach did not perform much better than the random baseline.

### Brain region identification

**Fig. 6:**
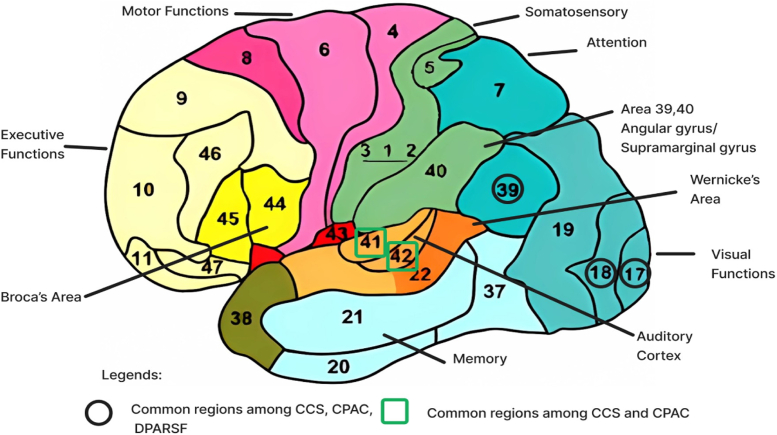
Shows the Brodmann's area.^59^ The different colours represent different brain functions. The common important brain regions from the Table 3 are highlighted.

The Integrated Gradients approach revealed a set of consistent brain connectivity patterns highly discriminative for the classification of ASD across the different preprocessing pipelines, one of which can be observed in Fig. 4. Important brain areas (parcellated per the AAL atlas), identified by the Integrated Gradients approach are shown in columns CCS, CPAC, and DPARSF in Table 3. The regions are arranged in descending order of importance score, with their corresponding Brodmann Areas.^58^ The most consistently important regions across pipelines are areas 17 (Primary visual cortex) and 18 (Secondary visual cortex) (Fig. 6). The thalamus (projection on medial geniculate body) which corresponds to Areas 41 and 42 (Primary Auditory cortex) showed some significance in the CCS and CPAC pipelines but were notably absent from the DPARSF results, suggesting less robust associations compared to the visual processing regions (Research Question 3).

All three pipelines rank the calcarine sulcus (BA 17) as the most important area. The cuneus (BA 17) emerges as a significant region. The pipelines also agree on the importance of the angular gyrus (BA 39), located in the posterior part of the inferior parietal lobe.

## Discussion

Regarding model performance, the 7% accuracy improvement from framewise displacement filtering aligns with autoencoders’ difficulty reconstructing samples with high head motion artifacts, underscoring the importance of motion correction in contrast to other studies.^40^ Motion correction ensures consistent biomarker detection, especially critical for younger populations where motion artifacts are more frequent, yet early diagnosis is most needed.^8^

Performance differences across preprocessing pipelines stem from methodological variations in spatial normalisation. NIAK normalises functional data to template space before calculating connectivity measures, while higher performing pipelines (DPARSF, CPAC, CCS) perform calculations in subject-native space before spatial normalisation. Preserving subject-specific neural signatures in native space explains superior classification performance of DPARSF, CPAC and CCS.

ABIDE I's multi-centre nature introduces site-specific noise from different scanners and protocols. While evaluating four distinct preprocessing pipelines partially addressed these variations, NIAK's lower performance suggests template-space normalisation could be more sensitive to cross-site heterogeneity. Future investigations could explore advanced domain adaptation or harmonisation strategies to mitigate site-based variability while maintaining native space analysis benefits.

Stable convergence in training curves provides insights into the model's learning process. During unsupervised pre-training, autoencoder reconstruction succeeds because the model reconstructs fMRI data without diagnostic labels, capturing fundamental neural patterns shared between groups. Supervised fine-tuning reveals how subtle, yet consistent, neurobiological differences are between individuals with ASD and with typical development. Once diagnostic labels are introduced, the network identifies these distinctions without overfitting. Two-stage training (first modelling shared neural representations, then identifying discriminative patterns) achieved high classification accuracy, consistent with multi-stage training findings in neuroimaging tasks.^60^

ROAR benchmarking provides critical insights into which interpretability methods are most reliable for functional connectivity data. Gradient-based methods demonstrated superior performance by identifying the most discriminative features for ASD classification, with Integrated Gradients emerging as the most reliable approach and LIME performed the same as the random baseline. These findings contrast sharply with the original ROAR study,^28^ which showed Integrated Gradients performing poorly and similarly to random baselines in image classification tasks and LIME performing well. The divergent results emphasise that optimal interpretability methods depend on both model architecture and data modality, highlighting the importance of domain-specific validation.

LIME's poor performance relative to gradient-based methods suggests perturbation-based techniques disrupt critical connectivity patterns in functional brain networks. Unlike image data where local perturbations isolate relevant features, functional connectivity represents holistic brain network interactions that are fundamentally altered when individual connections are perturbed. The superior performance of gradient-based methods in fMRI data can be attributed to the continuous, smooth nature of functional connectivity patterns, where gradient-based approaches preserve the holistic brain network interactions whilst tracing attribution signals through learned connectivity patterns. This aligns well with fMRI's temporal coherence and the distributed nature of neural processing, where information emerges from coordinated activity across multiple regions rather than isolated focal activations. Gradient-based methods succeed because they capture network–level relationships without disrupting underlying connectivity structure. However, ROAR limitations include high computational cost from repeated retraining and sensitivity to retraining variability, making it most practical for shallow models like our two-layer architecture.

Consistent identification of visual processing regions across all preprocessing pipelines provides compelling evidence for their central role in ASD classification. The convergence on primary visual cortex (calcarine sulcus, Brodmann area 17), secondary visual cortex (cuneus, Brodmann areas 17–18), and superior occipital gyrus represents a robust finding that transcends methodological differences in data preprocessing.

Brodmann area 17 (primary visual cortex) prominence in our results carries particular significance given its independent validation across multiple research domains. Genetic studies using completely different models and datasets have highlighted the importance of Brodmann areas 17 and 18 in autism,^18^ while neurophysiological research has documented global motion perception deficits^61^ and atypical gamma oscillations^62^ associated with primary visual cortex in ASD. Our model's identification of these regions corroborates growing evidence that fundamental differences in early visual processing play a crucial role in ASD pathophysiology, potentially influencing broader social and behavioural symptoms.

Cuneus consistency across pipelines aligns with recent twin studies showing reduced brainstem-cuneus connectivity in individuals with autism compared to their co-twins with typical development.^63^ These connectivity changes in the low-level visual pathway affect both social information processing and basic perceptual abilities, providing a neurobiological mechanism linking early sensory processing to higher-order social symptoms. Supporting evidence comes from rs-fmri and eye-tracking studies that have directly implicated the cuneus in social dysfunction in autism.^64^ This understanding suggests that visual processing assessments could serve as objective screening tools, particularly visual motion perception tasks that could be implemented in primary care settings. Environmental modifications addressing visual sensitivities could also be prioritised in treatment planning, as visual processing differences appear fundamental to the broader ASD phenotype.

Angular gyrus identification (Brodmann area 39) across all pipelines provides additional validation, given its well-established role in language processing and the known communication difficulties characteristic of ASD.^65^ The convergence of language-related and visual processing regions suggests ASD classification depends on a distributed network involving both sensory and higher-order cognitive systems. The relative prominence of visual processing regions over frontal areas in our findings aligns with the resting-state methodology, where executive functions requiring active planning would not be expected to drive classification.

We also identified associations of autism with temporal-frontal regions associated with auditory information processing (Brodmann area 21) and speech production (Brodmann area 44 and 45). While this association is not as strong as the visual cortex association it still sits well with that people with ASD are recognised to have difficulties processing auditory information.

Cross-pipeline consistency of these findings, combined with their alignment across genetic, neuroanatomical, and functional research approaches, provides strong validation that our model captured genuine neurobiological markers of ASD rather than dataset-specific artifacts. Visual processing regions’ robustness as discriminative features suggests they represent a fundamental neurobiological signature present across the autism spectrum.

While our interpretability methods identify which regions are most discriminative for classification, they do not specify connectivity directionality. Future work should incorporate signed connectivity analysis to determine whether identified regions exhibit hyper or hypo-connectivity patterns, which would provide more mechanistic insights into ASD pathophysiology.

Our high accuracy across all participants suggests the identified visual processing regions are discriminative regardless of severity level. This finding has important implications for understanding ASD as a neurodevelopmental condition with consistent underlying neurobiological signatures, despite its heterogeneous behavioural presentation. Future work should examine whether biomarker importance varies with symptom severity. However, while these regional findings offer valuable insights, ASD's complexity necessitates consideration of broader network-level interactions rather than isolated regional differences. Future investigations should focus on understanding how these visual processing regions interact with other brain networks to produce the full spectrum of ASD symptoms.

Notably, executive dysfunction represents a core feature of autism that our resting-state methodology may not fully capture. Individuals with prominent cognitive control difficulties may require complementary task-based fMRI protocols targeting executive networks. Future work should also investigate whether visual processing biomarkers remain discriminative during active task states and develop brief executive function protocols suitable for clinical implementation.

Our binary ASD versus control classification approach, while achieving high accuracy, may not capture the full spectrum of neurodevelopmental presentations. Future work should explore whether these visual processing biomarkers remain discriminative in more complex classification scenarios involving multiple neurodevelopmental conditions.

Several important limitations must be acknowledged regarding the generalisability of our findings. The ABIDE I dataset's gender imbalance (85.2% male) reflects diagnostic practices from the early 2010s when autism was believed to affect males at a 4:1 ratio.^66^ Current evidence indicates that females often mask ASD symptoms more effectively,^67^ leading to historical under-diagnosis. This gender bias in our training data may limit the model's sensitivity for detecting ASD in females, potentially perpetuating diagnostic disparities.

Similarly, the ethnic composition of ABIDE I may not reflect current understanding of ASD prevalence across diverse populations. While pre-2010 data showed lower diagnosis rates in certain ethnic groups, recent studies indicate higher prevalence rates among Black and Chinese children,^68^ likely due to increased awareness, improved diagnostic tools, and better access to services for minority populations.

Regarding age range, while our dataset (7–64 years) cannot support very early detection at age 2, it directly targets today's primary clinical need. ASD diagnosis has shifted dramatically. Since most new diagnoses now occur in adults, the ABIDE I dataset therefore addresses the most pressing current challenge: providing objective diagnostic support for adults who represent the majority of today's diagnostic demand.

These limitations highlight that our findings should be considered preliminary until validated in more demographically representative and contemporary datasets. Future work must prioritise testing these biomarkers in gender balanced and ethnically diverse populations to ensure equitable diagnostic applications. Additionally, the robust visual processing biomarkers identified here provide specific targets for intervention development, suggesting that addressing visual sensitivities and motion processing differences could have broader impacts on social and communicative symptoms in ASD.

This research significantly advanced ASD classification using deep learning on resting-state fMRI data. Our model achieved 98.2% accuracy using the DPARSF pipeline, surpassing previous benchmarks. We pioneered the use of ROAR to benchmark interpretability methods for functional connectivity data, identifying gradient-based approaches, particularly Integrated Gradients, as most reliable for this application. Our feature analysis consistently highlighted specific visual processing regions-primarily the calcarine sulcus and cuneus, along with the superior occipital gyrus-as critical biomarkers for ASD classification across different preprocessing pipelines. The angular gyrus, involved in language processing, was also consistently identified as significant. These findings aligned with independent genetic studies highlighting Brodmann areas 17 and 18 in autism, validating that our model captured genuine neurobiological markers rather than dataset-specific patterns. The consistency of these biomarkers across the dataset suggests they may represent a fundamental neurobiological signature across the autism spectrum. Our focus on visual processing biomarkers represents an attempt to identify neurobiological signatures of fundamental ASD features that transcend current diagnostic boundaries. Future diagnostic revisions may benefit from incorporating such biological markers, as neurobiological understanding could help improve current classification systems.

Despite promising results, several limitations must be acknowledged. Our binary classification approach may overlook characteristics shared with other neurodevelopmental conditions. While we report strong results in the ABIDE I dataset, testing on other ethnically diverse datasets or prospectively acquired clinical data is essential to confirm real-world applicability. Future work should develop models that highlight important regions on a per-scan basis rather than only analysing group differences, potentially enhancing clinical interpretability and trust.

## Contributors

SV conceived the core methodology, conducted the literature review, implemented the deep learning code, wrote all the manuscript drafts, and responded to reviewer feedback. KG assisted with data acquisition and preprocessing, assisted with the literature review, contributed to hyperparameter tuning, reviewed manuscript drafts and contributed to manuscript revisions. AA conceived the study and served as the academic lead of the project. He provided overall technical supervision, guided the interpretability analysis design, and refined the manuscript text. AW offered domain expertise on neuroimaging paradigms, helped interpret the model's decisions and contextualise results within ASD literature, and contributed to manuscript revisions. EI provided senior oversight of the research design, helped frame the clinical significance and framed the biomarker idea, and contributed to manuscript revisions. RS contributed clinical ASD expertise, validated the study's medical relevance with a medical literature review, and critically revised final drafts. SV, KG, and AA accessed and verified the underlying data. All authors read and approved the final version of the manuscript.

## Data sharing statement

The ABIDE I dataset used in this study is publicly available through the Preprocessed Connectomes Project at http://preprocessed-connectomes-project.org/abide/. The complete AI model with the preprocessing and analysis pipeline is available through our code repository at: https://github.com/v1dya/XAI-for-ASD.

## Declaration of interests

SV, KG, AA, AW, and EI declare no competing interests. RS declares grants from UK NIHR, Jazz IIS research grant, SBRI Healthcare Competition, Innovate biocatalyst CITADEL, Innovate UK bio-medical Catalyst award, Baily Thomas Charitable Fund, EPSRC Network, NHIR AI grant, Angelini IIS grant, ESRC, LivaNova, NHS England, Peninsula CRN, and Innovate Biocatalyst award, all paid to institution; honoraria for lectures, presentations, speaker bureaus, and organising educational events from LivaNova, UCB, Eisai, Veriton Pharma, Neuraxpharm, Bial, Angelini, UnEEG, TEVA, and Jazz/GW pharma, outside the submitted work; and unpaid participation on NICE Epilepsy board.

## Figures and Tables

**roar zoomed:**
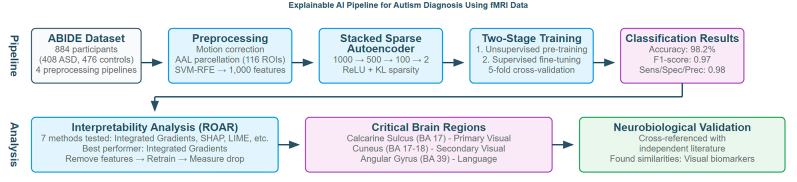
**roar zoomed in.png** Detailed ROAR (Remove And Retrain) analysis results showing the impact of progressive feature removal on model accuracy for all seven interpretability methods, demonstrating the superior performance of gradient-based approaches.

**model arch:**
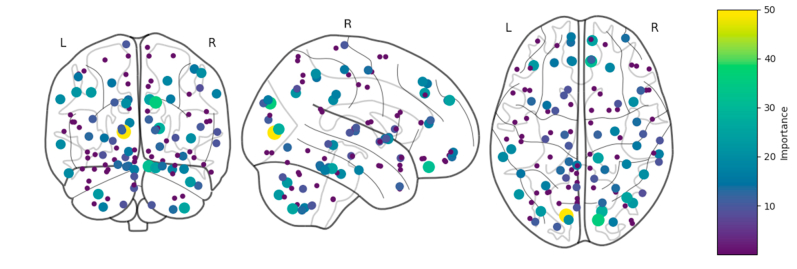
**model_arch.png** Detailed architecture diagram of the Stacked Sparse Autoencoder model, illustrating the complete pipeline from functional connectivity input through feature reduction to final ASD/TC classification.

**loss.png:**
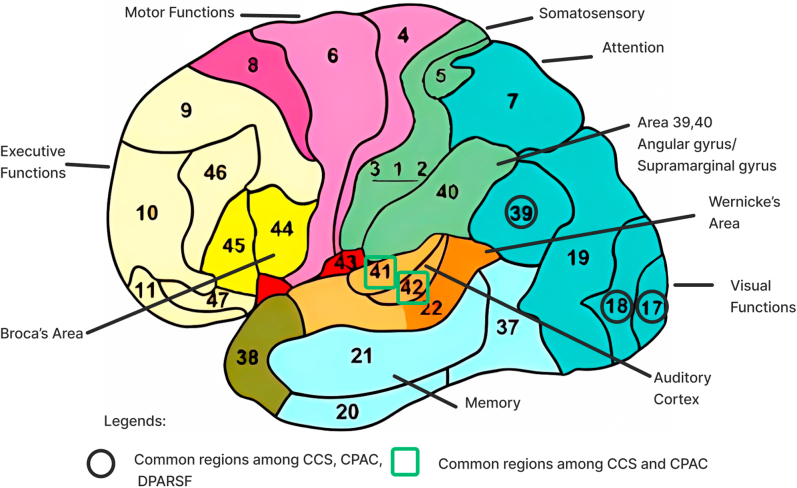
**loss.png** Training and validation loss curves across all model components (AE1, AE2, classifier) during the two-stage training process, showing stable convergence without overfitting during both unsupervised pre-training and supervised fine-tuning phases.

**flow diagram:**
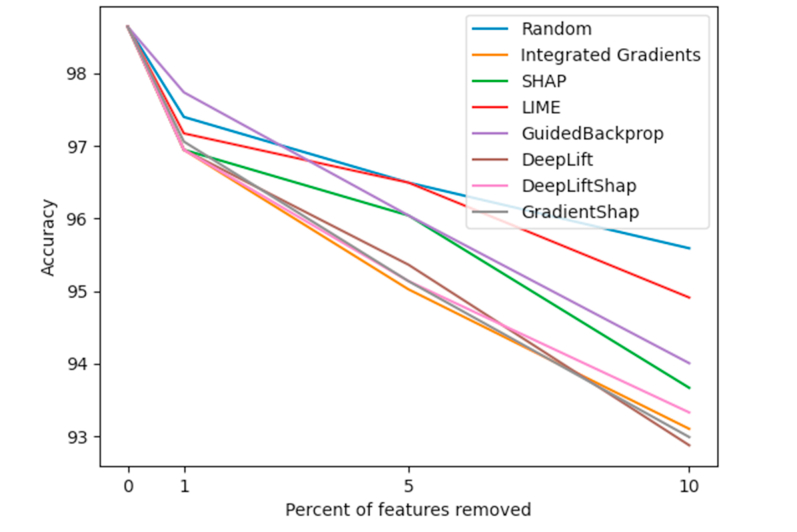
**flow diagram.png** Complete methodological pipeline showing data preprocessing, feature extraction, model architecture, interpretability analysis, and validation steps for explainable AI-based autism diagnosis using fMRI data.

**dparsf.png:**
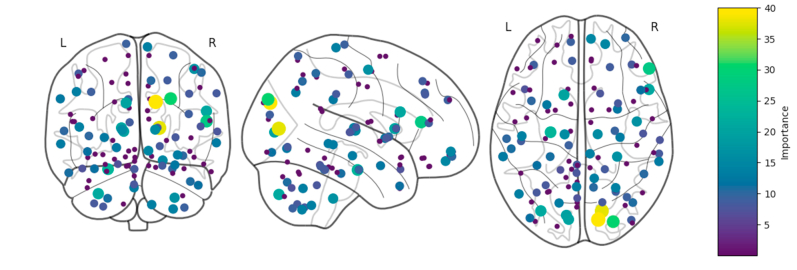
**dparsf.png** Important Regions of Interest (ROIs) identified by the Integrated Gradients approach for the DPARSF pipeline, with colour intensity indicating importance level. The three views show: posterior/back view (left), right lateral view (middle), and superior/top view (right).

**cpac.png:**
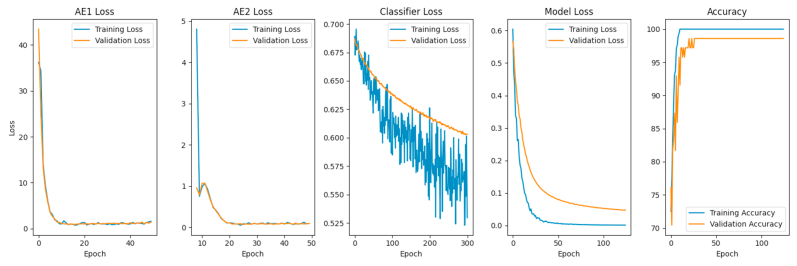
**cpac.png** Important Regions of Interest (ROIs) identified by the Integrated Gradients approach for the CPAC pipeline, with colour intensity indicating importance level. The three views show: posterior/back view (left), right lateral view (middle), and superior/top view (right).

**ccs.png:**
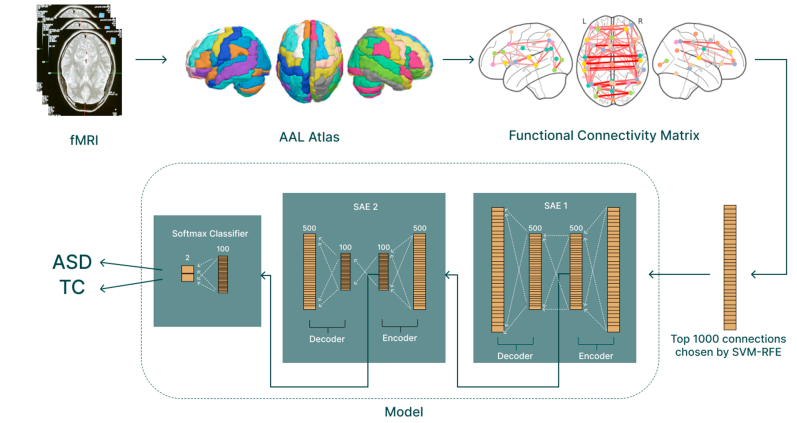
**ccs.png** Important Regions of Interest (ROIs) identified by the Integrated Gradients approach for the CCS pipeline, with colour intensity indicating importance level. The three views show: posterior/back view (left), right lateral view (middle), and superior/top view (right).

**Broadman_area.png:**
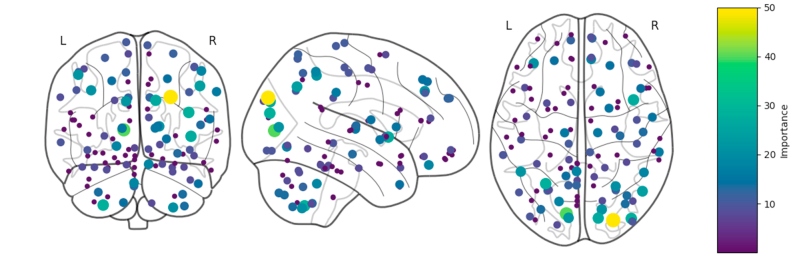
**Broadman_area.png** Brodmann's area map showing different brain functional regions with highlighted areas (17, 18, 39, 41, 42) that were consistently identified as important across preprocessing pipelines for ASD classification.
